# Cows’ Milk Allergy-Associated Constipation: When to Look for It? A Narrative Review

**DOI:** 10.3390/nu14061317

**Published:** 2022-03-21

**Authors:** Frances Connor, Silvia Salvatore, Enza D’Auria, Maria Elisabetta Baldassarre, Miriam Acunzo, Gaia Di Bella, Ilaria Farella, Simona Sestito, Licia Pensabene

**Affiliations:** 1 Department of Gastroenterology, Hepatology and Liver Transplant, Queensland Children’s Hospital, Brisbane 4101, Australia; adslbds0@tpg.com.au; 2 Mayne Academy of Pediatrics, Faculty of Medicine, University of Queensland, Brisbane 4101, Australia; 3 Department of Pediatrics, Ospedale “F. Del Ponte”, University of Insubria, 21100 Varese, Italy; silvia.salvatore@uninsubria.it (S.S.); dibellagaia01@gmail.com (G.D.B.); 4 Department of Pediatrics, Vittore Buzzi Children’s Hospital, University of Milan, 20154 Milan, Italy; enza.dauria@unimi.it (E.D.); miriam.acunzo@unimi.it (M.A.); 5 Department of Biomedical Science and Human Oncology, Neonatology and Neonatal Intensive Care Unit, “Aldo Moro” University of Bari, 70124 Bari, Italy; mariaelisabetta.baldassarre@uniba.it; 6 Department of Biomedical Science and Human Oncology, Clinica Medica “A. Murri”, “Aldo Moro” University of Bari, 70124 Bari, Italy; ilafarella@yahoo.com; 7 Department of Medical and Surgical Sciences, Pediatric Unit, University “Magna Graecia” of Catanzaro, 88100 Catanzaro, Italy; sestitosimona@unicz.it

**Keywords:** constipation, allergic disease, food allergy, cows’ milk allergy, children

## Abstract

Constipation is a very common disorder, mostly functional in nature, that may persist for years in up to 35–52% of children. Food allergy prevalence, severity and persistence are increasing over time, and cows’ milk protein is the commonest food allergen recognised to affect gastrointestinal motility in children. There is mounting evidence of the role of cows’ milk (CM) allergy (CMA) in children with constipation. With this narrative review, we aim to provide clinicians with an updated and critical overview of food allergy-associated constipation. We searched Embase, Medline and the Cochrane Library, using keywords related to the topic. Only reviews and studies including children aged 0–17 years that were published in English were considered. Constipation has been reported in 4.6% of infants with CMA; the prevalence of food allergy underlying chronic constipation in children resistant to conventional treatment and presenting to tertiary clinics ranges between 28% and 78%. The identification of predisposing risk factors and of a specific phenotype of food allergy-induced constipation remains elusive. No allergic tests, radiological or motility investigations achieve sufficient sensitivity and specificity to screen children for CMA-related constipation. A 4-week cows’ milk protein (CMP) elimination diet may be considered for children with chronic constipation resistant to conventional treatment and who lack alarm sign/symptoms of organic diseases. In subjects with ameliorated symptoms on CMP elimination, the diagnosis of CMA should be confirmed by a food challenge to avoid an unnecessary protracted diet.

## 1. Introduction

Constipation and food allergies are frequent concerns for parents of all age children consulting general and specialist health care providers [1,2]. The exact prevalence and the relation of these two combined disorders are currently uncertain [3,4]. Genetic, dietary, immune and motility factors have been described. However, no predictive and accurate biomarker or investigation for food allergy-associated constipation (FA-C) has been identified [4,5]. Thus, both under and over diagnosis of allergy may occur, causing persistent symptoms, possibly inappropriate prescription of diet or drugs, an impaired quality of life and an increased use of health care resources [6,7].

The aim of this narrative review is to provide clinicians with an updated and a critical overview of current knowledge on the possible association between constipation and food allergy, analyzing the weaknesses and/or the strength of published reports, to improve the diagnostic approach and the management of these patients.

Methods: the Cochrane Library, Embase and Medline were searched using the following key words: “allergy”, “allergic”, “hypersensitivity”, “reaction”, “intolerance”, “tolerance”, “allergic diseases”, “food allergy”, “allergic proctocolitis”, “atopic dermatitis”, “eczema”, “allergic rhinitis” “atopic disease”, “constipation”, “gastrointestinal motility disorders”, “food allergy associated constipation”, “dairy”, “milk”, “cow”, “cows”, “cow’s”, “cows’ milk allergy”, “cows’ milk allergy associated constipation”, “neuroimmune”, using “AND”/“OR” operators as appropriate. References cited in papers identified by database searches were also included. Abstracts were reviewed by 2 reviewers (FC and LP) and only studies and reviews including children (aged 0–17 years) and published in English were considered.

### 1.1. Childhood Constipation

Constipation is characterised by an infrequent and/or difficult passage of stools. It is a common disorder with a worldwide reported prevalence of up to 30% (median 8%) of the paediatric population [1,8]. There is no gender difference until adolescence, but there is a female predominance later on in life [9,10]. Constipation is the main complaint in 3% of all referrals to general paediatric outpatient clinics [11] and in 11–25% of visits to paediatric gastroenterologists [12,13]. In 35–52% of children with chronic constipation, symptoms persist for years [14], and in almost a third of subjects with functional constipation it continues into adult life [15,16].

Functional constipation (FC) is the most frequent diagnosis in children above one year with gastrointestinal disorders [8]. According to the Rome IV paediatric criteria, functional constipation is defined by the presence of two or more of the following symptoms for a minimum of a month: less than three motions per week; at least one episode of fecal incontinence per week after the acquisition of toileting skills; retentive posturing or excessive volitional stool retention; painful or hard bowel motions; the presence of a large faecal mass in the rectum or history of toilet obstruction by large stools. To fulfill the definition of functional constipation the symptoms cannot be explained by another medical condition after appropriate medical evaluation [17].

Environmental, behavioural, immunological and genetic factors may contribute to functional constipation [9,12,18,19,20,21,22,23,24,25,26,27,28,29,30,31,32,33,34,35,36,37,38,39,40,41]. The typical mechanism is behavioural stool withholding because of pain, inflammation or anal fissure, which can lead to a vicious cycle of large, firm, painful stools and further withholding [12,18,19,20,21,22]. Children with autism, neurological or motor disorders, developmental, psychiatric and behavioural conditions, atopy and food allergies and children exposed to abuse, violence or psychological stress are more prone to develop constipation [18,19,20,23,24,25,26,27,28,29,30,31,32,33,34,35]. Twin studies demonstrate that 59% of childhood constipation can be explained by genetic predisposition [36], although the exact genetic mechanisms are yet to be defined [9]. Of note, in allergy, atopy and autism, complex polygenic influences have been suggested [37,38,39,40]. Moreover, in autistic children a 50% prevalence of FC [23] and double the rate of food allergy has been reported compared to controls [41].

Several studies suggest an association between FC and abnormal gut microbiota, with probable bidirectional effects between gut transit and microbiota, through different cytokines and metabolites (involving bile acids, short-chain fatty acids, 5-hydroxytryptamine and methane) [42].

The mechanistic relationship between dysbiosis and constipation is still poorly understood, but altered microbiota may upregulate the expression of serotonin transporters, which are transmembrane transport proteins that re-uptake excessive 5-hydroxytryptamine involved in gastrointestinal motility, contributing to chronic constipation [43].

### 1.2. Cows’ Milk Allergy

Cows’ milk is the commonest food allergen recognised to affect gastrointestinal motility in children, mostly via non-IgE mediated reactions [44]. Cows’ milk allergy (CMA) affects 1–5% of children [44,45]. Up to 32–60% of cases report gastrointestinal symptoms, 5–90% have skin manifestations, while anaphylaxis affects 0.8–9% of patients [44]. Infants with reflux, irritability or diarrhoea may grow up to be school-aged children with constipation [46,47]. Persistence is more likely in children with IgE-mediated CMA or other atopic diseases, multiple food allergies or with an atopic parent [46,47].

Multiple risk factors for food allergy (FA) have been considered, including genetic, epigenetic and environmental factors, affecting barrier function (skin, gut) and immune pathways. These include race/ethnicity (non-white children at increased risk), gender, microbiota, drugs (i.e., antibiotics and acid inhibitors), hygiene, reduced exposure to siblings, day care and animals, vitamin D insufficiency, comorbidity (i.e., obesity, dermatitis), multiple dietary factors (such as reduced consumption of omega-3-polyunsaturated fatty acids, antioxidants or fibre), gastrointestinal infection and stress [45,48,49,50]. The microbiota appears to play a pivotal role in the development of food allergy. Recent studies in pre-clinical mouse models using targeted bacteriotherapy suggest that commensal bacteria activate a MyD88/ROR-γt pathway in nascent Treg cells to protect against FA. In contrast, dysbiosis impairs this regulatory response and promotes allergic disease [51,52]. In fact, the discovery of this mechanism indicates that not necessarily food proteins but microbiota proteins can induce allergy. Both diet- and host-derived metabolites generated by commensal bacteria regulate mucosal immune tolerance, and they promote epithelial barrier integrity [52].

Delayed diagnosis of CMA is reported, particularly in children with non-IgE mediated forms [47,53,54]. Undiagnosed CMA can compromise nutrition and growth, and it can reduce quality of life of both the patient and the family [30,55,56,57].

A gastrointestinal food allergy can be mediated by IgE, such as in gastrointestinal anaphylaxis and oral allergy syndrome, or, more commonly, by non-IgE mediated or mixed mechanisms, as in food protein-induced enterocolitis syndrome (FPIES), food protein-induced allergic proctocolitis (FPIAP), eosinophilic esophagitis (EoE) and eosinophilic gastrointestinal disease (EGID) [58]. Aside from these well recognised allergic phenotypes, non-IgE mediated CMA has been associated with a wide range of non-specific gastrointestinal manifestations, such as regurgitation, vomiting, food refusal or aversion, poor growth, colic, abdominal pain, diarrhoea and constipation [44,56,57,59,60,61]. Anal fissures and recurrent rectal prolapse have been reported in children with CMA-associated constipation [18,19,20,24,30,31,32,62]. As many of these symptoms are also present in reflux disease and functional gastrointestinal disorders, the distinction between allergic and non-allergic cases is often challenging [63,64]. However, it is vital to not miss an underlying diagnosis of CMA, as symptoms are amenable to dietary modification, and also because official guidelines on constipation management recommend invasive testing, ionizing radiation, mental health referral or surgery for intractable cases of constipation [3]. The “NICE guidance on Food allergy in under 19s: assessment and diagnosis (CG116)” recommends that food allergy be considered in all children with chronic symptoms of reflux or constipation, particularly in treatment-resistant cases [57]. The combination of persistent symptoms in different organ systems is particularly suggestive of allergy [51,61,63,65].

### 1.3. Cows’ Milk Allergy-Associated Constipation

There is increasing recognition that many gastrointestinal motility disorders may be provoked or exacerbated by food allergy [61,64,66,67,68,69,70]. Although CMA has been reported as the commonest cause of chronic constipation in infants [71], amongst infants with CMA, diarrhoea is actually more prevalent (61%) than constipation (4.6%) [72]. Some children with CMA switch from diarrhoea in infancy to constipation [47] around the time of toilet training [73]. Constipation is the most frequent delayed clinical manifestation of CMA [47]. The prevalence of food allergy underlying chronic constipation in children not responsive to conventional treatment and presenting to tertiary clinics ranges between 28–78% depending on age, inclusion and diagnostic criteria [18,19,20,24,25,26,27,28,29,30,31,32].

Shorter duration of breastfeeding and early exposure to CM may play a role in the development of constipation and anal fissure in infants and young children [74,75]. However, there is no evidence that constipated children consume more or less dairy than controls later in childhood [76]. In a Turkish study, three-day food records in children with functional constipation did not show a reduced fibre intake, but they did demonstrate a reduced protein intake. Whether this was due to reduced dairy was not reported [77].

Typically, children with constipation related to CMA have other manifestations of allergy or atopy (such as eczema or allergic rhinitis), or symptoms elsewhere in the gastrointestinal tract (such as regurgitation). Symptoms may be asynchronous, starting with infantile regurgitation or diarrhoea and progressing to constipation and dermatitis or respiratory manifestations years later [47]. Nonetheless, constipation can be the only manifestation of CMA [24,78]. In such cases, food allergy-associated constipation (FA-C) may present identically to functional constipation. Notably, unlike functional constipation, symptoms, including withholding behaviours, resolve on elimination of the offending food [20].

Approximately a fifth of children with CMA-related constipation have multiple food allergy [19,28,29]. The most frequent culprits are wheat, soy, corn, egg and rice [19,25,28,29,46,79,80]. Moreover, a plethora of foods have been implicated in food-allergic constipation, including tomato, fish, cocoa, goats’ milk, soy, oranges and legumes [19,80]. Double blind placebo-controlled food challenge (DBPCFC) protocols using oligoantigenic diet, either from the outset, or as a step up intervention for patients who did not respond to dairy elimination alone, detected an additional 8–24% of children whose constipation was related to food allergies [19,28,29]. Most of these children reacted to more than one food, including dairy in almost all cases. Reactions to exclusively non-dairy foods were uncommon, with a few children affected by egg allergy in one of the studies [29].

Constipation in food allergy has been classified as a non-IgE mediated manifestation, and testing for food-specific IgE, via skin prick, RAST or immune-CAP testing is uninformative [31,32]. Consequently, allergy tests are not recommended [3]. Rather than a generalized motility disorder, FA causes outlet type constipation. Transit studies show faeces are held up at the rectum, rather than due to impaired colonic propulsion [25]. In fact, colonic transit may be rapid as far as the rectum [81]. This pattern of “rapid transit constipation” is common in children with chronic constipation, occurring in 29% of 1000 transit studies in one series [82]. In a group of 55 patients with rapid proximal colonic transit, there was a family history of allergy in 10.9%, and symptoms suggestive of food allergy, including abdominal pain (80%), anal fissure (27.3%) and/or other allergic symptoms (e.g., asthma, eczema) in 43.6%. Of eighteen children treated with dietary exclusion, symptoms resolved in 50% [83]. Previously, barium studies have shown rapid intestinal transit in food-allergic patients [84,85]. In children with food allergy manifesting as constipation, transit studies normalise on elimination diet [25].

Multiple pathogenetic mechanisms may contribute to the outlet type constipation associated with CMA [4]. Inflammation, behavioural withholding and abnormal anal sphincter function may all contribute. Food allergy-related constipation is associated with proctitis, with increased eosinophils in rectal mucosal biopsies [18,19,24,25,26,28,73]. However, proctitis is absent in up to 40% [18]. Mast cell density and the proximity of mast cells to enteric nerves in rectal biopsies were found to be markers of food-allergic constipation [29]. Pain associated with abdominal cramps, proctitis, dyschezia, perianal excoriation or fissures may contribute to withholding behaviours [18,19,20,21,22,86] that resolve on elimination diet [20]. In addition, reversible anal sphincter dysfunction has been documented in FA-constipation. Anorectal manometry demonstrates elevated anal sphincter resting pressure and it may show impaired relaxation [25,28,29] mimicking Hirschsprung’s disease, but resolving on elimination diet [87,88,89,90,91]. Rectal biopsy is not indicated if symptoms respond to diet [87], but it remains the gold standard for exclusion of Hirschsprung’s in indeterminate cases. Both manometric and histological abnormalities are reversible with elimination diet in children with constipation due to food allergy [19,28,29].

Most children with constipation do not require investigations [3,17]. The internationally accepted Rome IV criteria for the diagnosis of functional constipation (FC), as listed above, allow a positive diagnosis to be based on a thorough clinical assessment, with a detailed medical history and a complete physical examination. This is usually sufficient to rule out most organic causes. Generally, it is unnecessary to perform any investigations before starting treatment. Red flag signs and symptoms suggesting organic causes must be excluded. These red flags are: neonatal onset of constipation (<1 mo), passage of meconium after 48 h of age, family history of Hirschsprung’s disease, ribbon-like stools, blood in the stools in the absence of fissures, failure to thrive, fever, bilious vomiting, abnormal thyroid gland, severe abdominal distension, perianal fistula, abnormal position of the anus, absent anal or cremasteric reflex, abnormal lower limb strength/tone/reflexes, tuft of hair over the spine, sacral dimple, deviated gluteal cleft, extreme fear during anal inspection and anal scars. Diagnostic procedures are recommended either in the presence of red flag symptoms/signs or in the case of a failure of first-line management (education, toilet training and conventional pharmacologic treatment, including regular use of high-dose osmotic laxatives and intermittent use of stimulant laxatives) [3,17].

Some variations in approach from country to country have been reported [92], however, indications and details of the different diagnostic strategies for chronic constipation are beyond the scope of this paper and recommended approaches are fully reported in dedicated consensus guidelines and expert reviews [3,34]. The controversies on when and how to investigate for FA among constipated children warrant a detailed literature review on the topic [3,34].

## 2. Critical Overview of Current Knowledge

### 2.1. CMA in Children with Constipation: A Literature Compendium

Various studies, dating back decades, have identified constipation as a symptom in some children with CMA. In a 1954 study of 206 infants with CMA, Clein found 10 (6%) with constipation [93]. Interest in the subject of cows’ milk allergy in constipation was renewed in the 1980s, when case reports emerged of constipation as the sole manifestation of CMA [78,94]. The hypothesis that CMA could cause constipation was first investigated prospectively by Iacono et al., who observed, in an open non-randomized study [24], that 21 out of 27 patients (78%) suffering from chronic constipation improved on a CMP elimination diet. The authors also reported that 15 out of 27 (71.4%) children with confirmed CMA (proven by elimination diet followed by oral food challenge) had positive beta-lactoglobulin-specific IgG and/or raised total IgE antibodies or peripheral eosinophilia [24]. A few years later, in a randomized, double-blind, placebo-controlled study, the same authors confirmed improvement of constipation in 68% out of 65 patients placed on CMP elimination diet and constipation relapse within 5–10 days of CMP reintroduction in all cases. The authors also found that this CMA-responsive population was more likely to have anal fissures and eosinophilic infiltration on rectal biopsy [18].

These two pioneering reports were followed by multiple studies with similar aims, most of which investigated the effect of CMP elimination diet in children whose constipation was medication-dependent or resistant [19,20,25,26,27,28,29,30,31,32,95,96]. Table 1 summarises the key results and characteristics of the prospective paediatric studies on this field.

Case numbers in each study were relatively small, which should be taken into account when interpreting the results. In some cases, food challenge was not performed. In others, some children who responded to dairy elimination did not relapse on dairy reintroduction. Whether this is due to the low dose of dairy used in challenges, the beneficial effects of participating in trials [95,99] or other factors is not clear. Notably, studies of CMA-induced constipation showed heterogeneity regarding the inclusion of a control group [18,20,30,32], laboratory tests [30,31,98] and elimination diet protocol of dairy or other foods [19,28,29]. In addition, studies did not include nutritional monitoring or analysis of the influence of psychosomatic diseases coexisting with CMA. All of these “controversies” are responsible for the ongoing debate on this topic.

However, focusing only on studies which used dairy challenge to confirm the diagnosis of CMA, overall, 285 of 469 (61%) of constipated children responded initially to dairy elimination, with relapse on dairy challenge in 46% [18,19,24,26,27,28,29,30,31,32,95]. Of note, there are few placebo-controlled trials due to the difficulties of masking dairy foods. Taking only those employing double-blind, placebo-controlled food challenges (DBPCFC), overall 102 of 216 children (47%) had challenge-proven dairy allergic constipation [18,19,28,29,95]. Response rates depend on research setting and inclusion criteria. In a community paediatric setting, none of 11 children with chronic, resistant constipation responded to dairy elimination [98]. By contrast, in a hospital based paediatric setting, the challenge-proven response rate to dairy elimination was 30% [95]. Challenge proven response rates were substantially higher in paediatric gastroenterology clinics, ranging from 28% to 78% [18,19,20,24,25,26,27,28,29,30,31,32]. The ESPGHAN/NASPGHAN guidelines recommend that children with medication-dependent or resistant constipation should be referred to paediatric gastroenterology clinics [3]. Thus, the high rates of dairy-responsive constipation detected in specialist settings may reflect patient selection.

In 2006, Iacono et al. evaluated anorectal manometry in patients with constipation and CMA. They found a lower critical volume when their rectum was distended by insufflation and a higher resting anal sphincter pressure compared to non-allergic children [28]. The authors first raised the hypothesis of intestinal inflammation induced by allergy to CMP in the pathogenesis of FA-C, causing outlet dysmotility, increased anal sphincter pressure, and eventual anal fissures. The latter can exacerbate faecal withholding behaviours, creating a vicious circle.

Neuroimmune interactions affecting motility have also been identified in the stomach of children reporting functional dyspepsia with a clear temporal association with allergen exposure [100]. Borrelli et al. documented a similar alteration in the rectum of children with constipation related to allergy. In particular, children with FA-C exhibited an increased density of mast cells in the rectal mucosa and a migration of these cells next to the submucosal rectal nerve endings, which were responsible for anorectal motility abnormalities. Both neuroimmune and histological changes resolved on elimination diet [29]. Turunen [27] demonstrated lymphonodular hyperplasia in the colon and in the terminal ileum, with high counts of γδ+ T cells in ileal biopsies in CMP elimination diet responders, which occurred in 34% of constipated cases.

**Figure 1:**
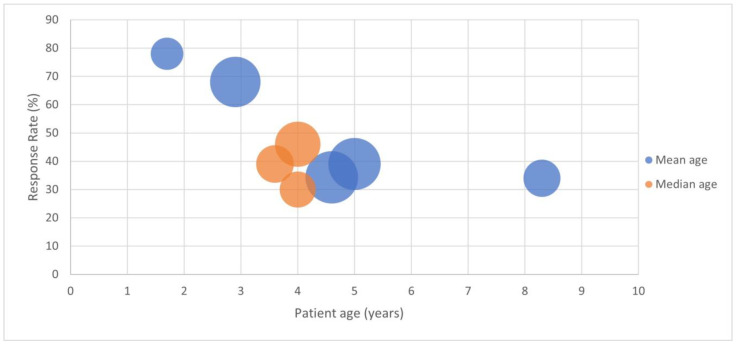
Response rates to CMP elimination diet and challenge in children with constipation, according to age of study participants, showing higher response rates in younger patients. Y axis indicates response to diet as a percentage. X axis indicates age of study participants in years, by measures of central tendency (mean age in blue or median age in orange). Bubble size indicates number of participants. Study details are in Table 1 above.

Although many studies identified higher rates of personal or family history of atopy or allergic diseases in responders to diet [18,19,27,29,47,79], this was not universal [20,31,80]. Children with FA-C also had a younger age of onset compared to non-allergic constipation [30], and younger children with constipation were more likely to respond to dairy elimination than older [20,95,101,102] (See also Figure 1). Tolerance was achieved in most cases within 6 months to two years [5,30,80].

In addition to the prospective trials of dairy free diet in children presenting with constipation, there are individual case studies and a small retrospective case series of children with known CMA in whom constipation was one of their CMP-induced symptoms. These include a small retrospective case series of 20 children referred to an allergy centre with various symptoms of CMA, focusing on the efficacy of a specific extensively hydrolysed formula. Symptoms in the 20 children included FPIAP and FPIES, and 5 had constipation. All responded to CMP-elimination, with 3 (60%) responding to eHF and 2 (40%) requiring amino acid based formula for resolution of constipation [103]. Another, earlier case series of 12 children with CMA-induced constipation (age range of 15 months to 11 years, mean 4.8 years) highlighted the fact that many had a history compatible with CMA in infancy, without having received a formal diagnosis of CMA in early life. Symptoms recalled by parents from infancy included vomiting, diarrhoea, irritability and poor growth [73].

Children with CMA-associated constipation have also been identified in studies evaluating investigations for CMA against elimination diet and challenge as the gold standard. Four studies assessing atopy patch tests, alone or in combination with other tests, for the diagnosis of CMA identified children with constipation as a manifestation of CMA [104,105,106,107]. These studies did not focus on constipation, and children typically had additional symptoms suggestive of CMA. With one exception [106], these studies excluded children with eczema/atopic dermatitis. These studies are summarized in Table 2. Apart from these four studies, other publications reporting on atopy patch tests for CMA have not specifically recorded constipation as a symptom [108,109,110,111,112,113,114,115,116,117,118,119].

In 2014, the ESPGHAN/NASPGHAN guidelines concluded that evidence for CMA in constipated children was conflicting, and it suggested an optional 2–4-week CMP elimination diet trial after a specialist gastroenterology review [3]. However, this document did not include all of the available evidence published at the time [19,25,26,27,28,29,32,95] because of multiple elimination diet in some cases [19,28,29] or a lack of a double-blind food challenge [26,27]. The included study with DBPCFC [46] was from an allergy centre, so was noted to possibly overrepresent the prevalence of the association [3]. Conversely, the conflicting evidence was mostly based on a single study showing a lack of response to CMP elimination diet in 11 medication-resistant constipated children [98], as well as the heterogeneous laboratory and clinical features in 4 other included studies, which had shown dairy response [18,24,30,31].

In 2014, Miceli Sopo et al. [120] reviewed 10 studies, with a total of 505 patients enrolled, consistently showing a benefit of CMP elimination diet in a percentage of constipated children, varying from 28 to 78% [5]. In contrast to the ESPGHAN-NASPGHAN guidelines, the authors asserted that the available scientific evidence was sufficient to formulate “a grade A recommendation”. In the absence of distinct clinical or laboratory features in the CM responder group, CMP elimination diet was proposed as an additional option for first-line therapy in chronic constipation [120].

In the last ten years, more evidence has accrued on the response to CMP elimination diet in a proportion of children with chronic constipation. There are now a total of 14 prospective studies from 8 countries prospectively evaluating the relationship between CMA and constipation. All but the Simeone study [98] have shown a response to CMP elimination diet in a proportion of children with chronic constipation [5,18,19,20,24,25,26,27,28,29,30,31,32]. In 2021, Mohammadi Bourkheili et al. conducted an open-label clinical study in 72 children (aged 4–14 years) suffering from functional constipation (according to Rome III criteria) who had not improved with at least 3 months of laxatives. Subjects received polyethylene glycol (PEG; 1 gr/kg/day) and high-fibre foods (at least 10 gr/day), and they were randomized to CMP elimination diet or no dietary restrictions for four weeks. At recall, significant differences were found between the CMP elimination diet and control groups in terms of resolution of constipation (71.4% vs. 11.4%), large stools reported by parents (25% vs. 53.6%) or detected during examination (17.1% vs. 50%), painful defecation (18.2% vs. 55.6%), retentive posturing (10% vs. 46%), frequency (≥1 episode/week) of faecal incontinence (25% vs. 50%, *p* = 0.001), infrequent (≤2/week) defecation (17.4% vs. 52.3%) and toilet obstruction (22.2% vs. 52.3%) [20]. Also in 2021, Gelsomino et al. emphasised the need to identify children at high risk for CMA as a cause of constipation. These include children of pre-school age, with a personal or family history of atopy and those with a previous diagnosis of CMA. In such children, the authors proposed the CMP elimination diet as the first line treatment for apparent functional constipation [96].

### 2.2. Diagnostic Work-Up for Food Allergy-Associated Constipation

Despite the ongoing debate within the paediatric gastroenterology community, constipation is listed as a ‘common’ gastrointestinal symptom of CMA in some recent national guidelines [61,121]. All major European and international allergy and paediatric gastrointestinal guidelines (BSACI, DRACMA, EACI, ESPGHAN) mention constipation as a possible allergic manifestation [44,61,63,121,122,123,124,125]. However, the pleomorphic nature of CMA and the heterogeneity of the studied populations accentuate the lack of a specific phenotype of patients with FA-constipation. A variety of invasive and non-invasive tests have been evaluated; however, none have proven accurate to detect children with FA-C.

In the absence of inflammatory bowel disease, ileal and colon lymphoid nodular hyperplasia has been associated with FA in children [27,99]. However, LNH is absent in a significant proportion of cases with FA-C [27,99]. Similarly, mucosal eosinophilia has been found in FA-C [18,19,27], but it is not present universally. Both findings improve on elimination diet in children with GI symptoms due to FA [19]. Nevertheless, invasive tests such as sigmoidoscopy or colonoscopy are unsuitable as diagnostic tests for food allergy in constipated children [126].

Conversely, faecal calprotectin is a non-invasive marker of gastrointestinal inflammation as it correlates with the level of mucosal inflammation [127,128,129]. It is currently used in inflammatory bowel disease. However, it has not proved to be useful in FA. A recent review concluded that despite elevation of faecal calprotectin levels in some infants [130,131] and older children [132] with CMA, there was no significant change in faecal calprotectin levels after challenge, and the specific cut-off values for allergic disease and FA-C remained indefinable [133].

Similarly, fecal biomarkers derived from eosinophils, such as eosinophil derived neurotoxin (EDN) and eosinophilic cationic protein (ECP), have been evaluated as possible biomarkers of non-IgE mediated food allergy but very limited data support their role in populations with gastrointestinal disorders [106,134,135]. Increased baseline mRNA levels of some cytokines (IL-13 and IL-10) in combination with faecal calprotectin and EDN have been reported as predictive of a positive food challenge outcome in a small study of children with both IgE and non-IgE mediated reactions. In future, these markers might serve as prognostic markers for symptomatic, IgE-mediated food allergy, but they need further validation in a larger patient cohort [135].

The atopy patch test (APT) has been considered for the diagnosis of non-IgE mediated food allergy and late phase reactions mediated by T lymphocytes [113]. Syrigou et al. applied APT in children with constipation, showing a positive correlation with a clinical response to dietary eliminations. Interestingly, wheat was found to be the main allergen diagnosed using APT in chronic constipation, followed by egg and milk [80]. However, these results have not been reproduced, and APT has fallen out of favor in some studies due to poor sensitivity. A recent meta-analysis found a pooled sensitivity of 44.2% and 86.9% specificity of APT for detection of CMA in children [136]. Studies focusing on gastrointestinal disorders, including constipation, have reported even lower sensitivity values [107]. As such, the role of APT in clinical practice remains controversial [80,104,105,107,137,138], and they are not currently recommended [63,139].

The allergen-specific lymphocyte stimulation test (ALST) is a promising tool in paediatric populations with non-IgE-mediated food allergies [140]. However, prolonged incubation times (up to 5 days) and large blood sample volumes limit its utility in children [134,141]. Although recently developed variation on traditional ALST allows shorter incubation times and lower blood sample volumes, this test still requires validation for the diagnosis of non-IgE mediated FA in children [142].

In the absence of reliable laboratory tests, an allergy-focused history and elimination with open food challenge remain the recommended diagnostic steps for dairy allergic constipation [143]. The UK NICE guidelines recommend this approach in all children with chronic GI symptoms, including constipation [57] and have used this strategy as a health care quality indicator since 2016 [144].

The Cows’ Milk-Related Symptom Score (CoMiSS) has been recently developed as an awareness tool in infants. It is based on quantification of crying, regurgitation, respiratory and skin symptoms, and stool consistency according to the Bristol scale, creating a combined score from 0 to 33. The authors’ ambition was to create an easy and an accurate tool that would help in the early recognition of CMA and, possibly, the evolution of symptoms during a diet intervention [145,146]. The original proposed cut-off of 12 was found to be highly predictive of the response to CMP elimination diet and the reaction at challenge in a number of studies, and it clearly discriminated symptomatic infants from controls [147,148]. Recently, Calvani et al. reviewed the current literature on CoMiSS and concluded that although it can already be considered a useful screening tool, it is not a substitute for elimination diet and food challenge for the diagnosis of CMA. This score requires further validation in different target populations, and optimal cut-offs need to be identified [134].

Families of older children with CMA constipation often describe a history of multiple symptoms in infancy and early childhood [46,47,79], including irritability, formula intolerance, colic and regurgitation, similar to those listed in the CoMiSS for infants [73,79,149]. As such, questions about the child’s health in infancy an important component of the allergy-focused history in a child with constipation. A clinician’s guide to allergy-focused history has been proposed by the Royal College of Paediatrics and Child Health in the UK [150]. It contains different modules for allergy screening of children as well as questions for children with suspected food allergy, focusing particularly on clinical manifestations rather than established diagnoses (e.g., itchy rash in skin creases rather than eczema).

The ESPGHAN diagnostic and therapeutic guidelines do not recommend routine testing for CMA in children with functional constipation [3,63], but the constipation guidelines suggest consideration of a CMP elimination trial for 2–4 weeks after failure of first line therapy (diary, education, toilet training, oral medication) and after excluding other underlying organic causes of constipation [3]. No guidance is given on how to select children for CMP elimination diet and there is no compulsion to implement it. A missed opportunity to diagnose CMA at this point poses a significant risk of inappropriate tests and treatments. These may involve biopsies, ionizing radiation, bowel preparation, colonoscopy, manometry and stoma surgery or resection [3]. Traditionally, CMP elimination diet has been offered to children based on high-risk features in the history, such as a personal history of prior food allergy, atopy or a family history of allergy [25,29,68,96]. However, some children with CMA constipation lack any such risk factors. Therefore, given the high pre-test probability of CMA in children with chronic constipation, some have advocated for the consideration of elimination diet trial in all children with chronic [120] or medication-resistant constipation [20,26,27,32,79,126]. Most trials have used a four-week elimination diet [19,20,24,26,27,28,30,32,98]. Improvement of constipation has been reported within days of CM elimination, regardless the presence of specific IgE towards cows’ milk proteins. Similarly, relapse usually occurs after 1–2 weeks of reintroduction of dairy product. Thus, avoidance of cows’ milk protein for a minimum of two to four weeks must be considered in children with chronic intractable constipation [3,5,151].

Taking all of the evidence into account, a 4-week CMP elimination diet could be considered not only in constipated children at high-risk of CMA (based on a personal history of atopy, prior food allergy or a family history of allergy), but also in all children with chronic constipation and/or unexplained outlet obstruction resistant to conventional treatment, absent any alarm sign/symptoms, before performing other diagnostic investigations (such as transit studies).

As with the other non-IgE mediated FA, a formal diagnosis of CMA constipation is made by CM elimination and challenge. If the symptoms disappear during CMP elimination diet and reappear with reintroduction, a causal link can then be established [44,152]. Dairy elimination can therefore be done for diagnostic or therapeutic purposes. Improvement and relapse of symptoms is usually within days of elimination and challenge [24,26,31]. However, symptom onset can be delayed by two to four weeks [29,47]. Due to the delayed onset of constipation symptoms, an in clinic/hospital challenge is not required in most cases. However, a challenge may be initiated in hospital if there is high risk of severe acute reaction. After confirming CMA as the cause of constipation with the 4-week CMP elimination diet and challenge, the CM can be eliminated for therapeutic purposes and then gradually reintroduced as tolerated [61,153].

## 3. Food Allergy-Associated Constipation Management

Current guidelines for constipation recommend education, behaviour modifications, adequate physical activity, a normal-for-age intake of fibre and fluids, progressing if needed to pharmacological treatments [3]. Elimination diet is commonly used as an add on treatment to conventional laxative and behavioural therapies [20,27,29,30,31,32], although CM avoidance diet solely may lead to symptom relief, especially in younger infants.

Overall, the quality of evidence for efficacy of laxative therapy in children is low, due to sparse data, a high risk of bias, the clinical heterogeneity of subjects and treatment and a short follow up in the available studies [154,155]. Nevertheless, the use of laxatives in clinical paediatric practice is well established [156], both for initial faecal disimpaction, if required, and subsequently for maintenance therapy [3]. Unfortunately, paediatric constipation often requires prolonged laxative treatment [36,157], including regular use of high-dose osmotic laxatives and intermittent use as rescue therapy of stimulant laxatives as part of the conventional therapy for functional constipation.

**Figure 2:**
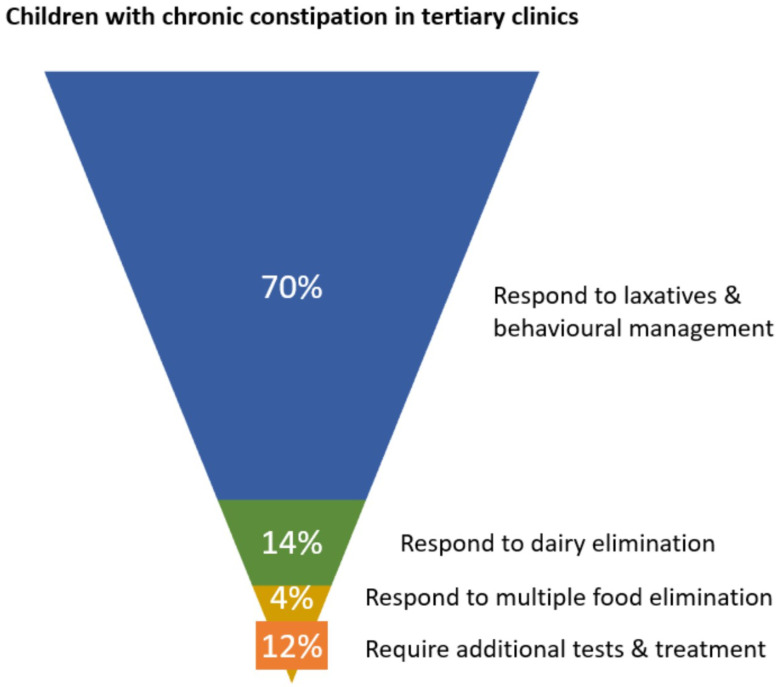
Schematic showing proportions of children in tertiary clinics responding to initial phases of management for chronic constipation.

Of children seen in tertiary clinics for functional constipation, approximately 70% experience symptom control on laxatives, leaving 30% medication-resistant [15,16]. Using figures from the DBPCFC studies showing response to CMP elimination diet in medication-resistant cases, a further 47% of children achieved treatment response while dairy free [18,19,28,29,95]. An expanded elimination diet, removing soy and egg in addition to dairy detected 12% with non-dairy allergies [29]. These outcomes are illustrated in Figure 2.

For infants requiring CM elimination, all guidelines on the management of CMA recommend continued breast feeding, with the mother avoiding all dairy products, as the ideal nutrition [44,61,63,121,123,124,125,143,158,159,160,161]. A maternal CMP elimination diet during breast feeding requires close supervision and support to protect the nutritional safety of both mother and baby, in accordance with published guidelines [122]. When human milk is not available or it is insufficient, extensively hydrolysed CM formula, rice hydrolysate or amino acid formula are recommended for the management of CMA [143]. Hypoallergenic formulas may vary for protein source and content, method and degree of hydrolysis and additional components, which affect tolerance and efficacy [143,162]. In formula-fed infants with food-allergic motility disorders, the use of extensively hydrolysed formulas has been shown to be effective [162]. Amino acid-based formula can be used if symptoms persist on extensively hydrolysed cow’s milk protein-based formula or if GI symptoms are associated with malabsorption and faltering growth [163]. This may also be required in infants whose symptoms were present while breastfed, if feeding with hydrolysed formula failed [164]. In the countries where soy formula is available, it may be considered for infants with CMA older than 6 months [44,61,63,123,124,165,166,167]. However, soy may exacerbate GI manifestations, as concomitant allergy with CMA has been reported in 10–15% of cases [168]. Rice hydrolysate formula is also a suitable alternative to CM-based hydrolysed formulas. Other plant-based milk alternatives are not nutritionally complete, and they are contraindicated as cows’ milk substitutes in infants [143,162].

The direct effect of hydrolysed proteins on gastrointestinal motility has been considered. A number of studies indicated that source protein (casein vs. whey) and degree of hydrolysis (intact protein vs. partial or extensively hydrolysed protein) may influence gastric emptying time [162,169]. Conversely, very limited data have been published on the effect of different proteins on intestinal motility. The modulatory effects on colonic motility of bovine whey and casein milk protein hydrolysates were tested in an ex vivo rat model [170]. Intestinal propagation frequency was decreased by casein protein hydrolysate, increased by a combination of 60% whey to 40% casein hydrolysate and unaffected by intact whey proteins [170].

No difference in stool consistency and frequency was found when a partially hydrolysed CM formula (hydrolysed whey/intact casein = 63/37) was used in healthy term infants [171] compared to an intact CMP formula (whey/casein = 61/39). Both groups of formula-fed infants had significantly fewer evacuations per day than breastfed infants. The colour and the volume of the stools of infants fed the hydrolysed formula resembled those of breastfed infants [171].

Most children with FA-C respond to elimination of CMP alone. Step up to oligoantigenic diet only benefits a minority. Step up to multiple food elimination may be considered in non-responders to CMP elimination, if justified by the severity or persistence of symptoms, particularly in young children with atopic comorbidities. A multiple food elimination diet requires close medical and dietetic support to optimize adherence and nutrition [172,173].

In addition to relieving current symptoms, the identification and the treatment of CMA may be related to long-term outcomes. A number of studies suggest a possible role of CMA in the development of functional gastrointestinal disorders (FGID) later in life [4,174,175,176,177,178]. The nature of this association remains to be evaluated.

Interestingly, Nocerino et al. found that dietary intervention with an extensively hydrolysed casein-based (EHCF) formula enriched with LGG in infants with CMA could influence the subsequent development of FGID at preschool age. A significant difference between the EHCF and the EHCF+LGG groups was observed for the rate of functional dyspepsia, abdominal pain and constipation (incidence rate ratio 0.44, 95% CI, 0.17–1.06; *p* = 0.07). The authors suggested that specific strain of probiotics may induce a beneficial epigenetic regulation of immune and non-immune gene expression of gut microbiota structure and function with increased production of the short-chain fatty acid butyrate [179]. In addition to accelerating the rate of resolution of CMA [180,181,182], this molecule may regulate gastrointestinal motility and pain perception, which are pivotal factors for the development of FGIDs [179].

Families should be advised that CMA tends to remit with time in most cases [30,61,63,80]. In children with multiple foods contributing to constipation, each should be challenged separately [80]. In most cases, FA-C improves after 6–24 months [30,80]. However, occasionally it persists into adult life [183].

Ideally all diets should be monitored and implemented by trained dieticians. For diagnostic purposes simple, time-limited, single food elimination diets in well-nourished children can be administered effectively from the paediatric gastroenterology or allergology clinic, using appropriate verbal instruction and written patient information. However, children who prove to have CMA and who require ongoing allergen elimination need supervision by an appropriately qualified dietician to ensure adequate intake of micro and macronutrients, including calcium and protein [44,61,63,184,185,186,187]. A recent study suggested that compared to healthy controls, patients with FA who lack confidence in FA issues and those following an uncontrolled, restrictive elimination diet are more prone to food aversion and eating disorders [188]. Conversely, another recent study reported an increased risk of eating disorders in children who had a previous history of chronic diarrhoea or constipation [189]. However, the possible relationship of the observed gastrointestinal symptoms to food allergy was not explored. Eating disorders themselves are commonly associated with gastrointestinal problems, including constipation [190]. Therefore, elimination diets require careful implementation with nutritional counselling and regular monitoring of growth and of compliance to ensure safety [44,61,63,188].

## 4. Conclusions

Constipation is the most frequent gastrointestinal disorder in children. Laxatives and behavioural approaches remain the cornerstone of therapy for functional constipation. However, many children present chronic symptoms requiring prolonged treatment. Challenges of adherence, parental compliance, additional investigations, and repeated consultations generate high health and economic impacts. Alarm signs and symptoms for organic disease should be excluded in all children with chronic constipation as a first step. Despite the ongoing debate due to some controversies on available data, there is mounting evidence for a role of CMA, particularly in constipated infants and young children with concomitant atopic signs and/or symptoms, or in outlet-type chronic constipation, unresponsive to conventional treatment. In this population, a 4-week CMP elimination diet as a diagnostic approach averts unnecessary invasive testing or prolonged treatment of constipation with drugs that require adherence and continuity. A number of studies have shown a beneficial effect of elimination diet and relapse of constipation at challenge in a large proportion of children. Nutritional assessment should be ensured to avoid macro and micronutrient deficiencies during the avoidance diet. Appropriate management for CMA avoids unnecessary suffering, averts inappropriate tests and treatments and conserves health care resources.

## Figures and Tables

**Table 1:** Prospective clinical trials of cows’ milk protein elimination diet in childhood constipation. Adapted with permission from ref. [97].

Year	Author	Country	Number of Cases	Study Type	Age Range (years)	Mean ($\bar{x}$) or Median Age (years)	Responders to Dairy Elimination (%)	Response Confirmed with Challenge (%)	Treatment Dependent or Resistant	Setting (Primary Care, Secondary, Gastro)
1995	Iacono G. et al. [24]	Italy	27	Prospective, open challenge	0.5–3	1.7 $\left(\bar{x}\right)$	78%	78%	either	ped GE
1998	Iacono G. et al. [18]	Italy	65	Prospective crossover RCT DBPCFC	1–6	2.9 $\left(\bar{x}\right)$	68%	68%	resistant	ped GE
1999	Shah N. et al. [25]	United Kingdom	14	Prospective	0.5–6.7	3.1 (med)	79% *	n/a	resistant	ped GE
2001	Daher S. et al. [26]	Brazil	25	Prospective, open challenge	0.25–11	nr	28%	28%	resistant	ped GE
2004	Turunen S. et al. [27]	Finland	35	Prospective, open challenge	3–15	8.3 $\left(\bar{x}\right)$	83%	34%	either	ped GE
2005	Carroccio A. et al. [19]	Italy	52	Prospective with DBPCFC	$\left(\bar{x}\right)$ 4.25+/−1.5 SD	4 (med)	46% *	46%	resistant	ped GE
2006	Iacono G. et al. [28]	Italy	36	Prospective with DBPCFC	0.75–10	3.6 (med)	39% *	39%	resistant	ped GE
2008	Simeone D. et al. [98]	Italy	11	Prospective	nr	nr	0	n/a	resistant	primary
2009	Borrelli O. et al. [29]	Italy	33	Prospective with DBPCFC	1–10.8	4 (med)	30% *	30%	resistant	ped GE
2009	El-Hodhod M.A. et al. [30]	Egypt	27	Prospective, long follow up	0.7–4	nr	78%	78%	resistant	ped GE
2010	Irastorza I. et al. [31]	Spain	69	Prospective, open challenge	0.5–14	5 $\left(\bar{x}\right)$	51%	39%	either	ped GE
2012	Dehghani S.M. et al. [32]	Iran	70	Prospective, case controlled RCT	1–13	4.6 $\left(\bar{x}\right)$	80%	34%	resistant	ped GE
2013	Crowley E.T. et al. [95]	Australia	30	Prospective crossover RCT with DBPCFC	1.5–12	nr	81%	33%	resistant	secondary
2021	Mohamma-di Bourkheili A. et al. [20]	Iran	35	Prospective case controlled RCT	4–14	5 $\left(\bar{x}\right)$	71%	n/a	resistant	ped GE

* Stars indicate studies where additional children were found to be allergic to other foods (commonly wheat, egg, soy, corn) or to multiple foods including dairy, manifesting as constipation. Setting: ped GE = paediatric gastroenterology center; primary= primary care; secondary=secondary care (hospital-based paediatric referral centre). nr = not reported. $\bar{x}$ = mean, med = median.

**Table 2:** Studies on investigations for detection of cows’ milk allergy in which constipation was reported by some participants.

Author	Year	Age Range (months)	Number in Study	Number with Constipation Prior to CMP-Free Diet	Constipation Responders to Dairy Free Diet/Challenge: Number (%)
De Boissieu, D. et al. [104]	2003	2–57	35	4	3 (75%)
Cudowska, B. et al. [105]	2010	6–144	28	7	3 (43%)
Kalach, N. et al. [106]	2013	1–18	25	5	2(40%)
Mowszet, K. et al. [107]	2014	3–36	39	not reported	2

## Abbreviations

AD, atopic dermatitis; AAF, amino acid formula; ALST, allergen-specific lymphocyte stimulation test; APT, Atopy patch test; BSACI, The British Society for Allergy & Clinical Immunology; CoMiSS, Cows’ Milk-associated Symptom Score; CM, cows’ milk; CMA, cows’ milk allergy; CMP, cows’ milk protein; DBPCFC, double-blind placebo-controlled food challenge; EAACI, European Academy of Allergy and Clinical Immunology; DRACMA, The World Allergy Organization’s (WAO) Food Allergy Special Committee’s Diagnosis and Rationale for Action against Cow’s Milk Allergy (DRACMA) guidelines; EAACI, European Academy of Allergy and Clinical Immunology; ECP, eosinophilic cationic protein; EDN, eosinophil derived neurotoxin; eHF, extensively hydrolysed formula; EHCF, extensively hydrolysed casein-based formula; EGID, eosinophilic gastrointestinal disorders; EoE, eosinophilic oesophagitis; ESPGHAN, European Society for Paediatric Gastroenterology Hepatology and Nutrition; FA, food allergy; FA-C, food allergy-associated constipation; FC, functional constipation; FGID, functional gastrointestinal disorder; FPE, food protein-induced enteropathy; FPIAP, food protein-induced allergic proctocolitis; FPIES, food protein-induced enterocolitis syndrome; IgE, immunoglobulin E; LNH, lymphoid nodular hyperplasia; NASPGHAN, North American Society for Paediatric Gastroenterology Hepatology and Nutrition; OFC, oral food challenge; RCT, randomized controlled trial.
